# [*N*′-(1,3-Dioxoindan-2-yl­idene)-2-oxido­benzohydrazidato-κ^3^ *O* ^2^,*N*,*O*]tripyridine­nickel(II) pyridine solvate

**DOI:** 10.1107/S1600536808023489

**Published:** 2008-07-31

**Authors:** Wei-Sheng Liu, Da-Xiang Wu, Jia-Yu Chen, Hui-Juan Wang, Xiao-Liang Tang

**Affiliations:** a Department of Chemistry, State Key Laboratory of Applied Organic Chemistry, College of Chemical Engineering, Lanzhou University, Lanzhou 730000, People’s Republic of China; b State Key Laboratory of Coordination Chemistry, Nanjing University, Nanjing 210093, People’s Republic of China

## Abstract

In the title compound, [Ni(C_16_H_8_N_2_O_4_)(C_5_H_5_N)_3_]·C_5_H_5_N, the Ni^II^ atom is six-coordinated by two O atoms and one N atom from the Schiff base ligand and by three N atoms from three pyridine mol­ecules, forming a distorted octa­hedral geometry. The Ni—O(phenolate) bond [1.9750 (16) Å] is shorter than the Ni—O(carbon­yl) bond [2.0840 (16) Å] and the Ni—N bonds (mean 2.120 Å).

## Related literature

For related Schiff-base structures, see: Qiu, Fang *et al.* (2006 ▶); Qiu, Luo *et al.* (2006 ▶); Qiu *et al.* (2007 ▶). 
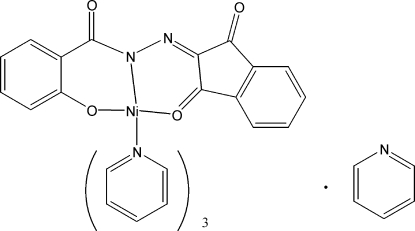


## Experimental

### 

#### Crystal data

[Ni(C_16_H_8_N_2_O_4_)(C_5_H_5_N)_3_]·C_5_H_5_N
*M* *_r_* = 667.35
Orthorhombic, 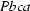

*a* = 17.1945 (13) Å
*b* = 17.6887 (13) Å
*c* = 21.4633 (16) Å
*V* = 6528.0 (8) Å^3^
*Z* = 8
Mo *K*α radiation
μ = 0.64 mm^−1^
*T* = 273 (2) K
0.19 × 0.18 × 0.15 mm

#### Data collection

Bruker SMART 1000 CCD area-detector diffractometer
Absorption correction: multi-scan (**SADABS**; Sheldrick, 1996 ▶) *T*_min_ = 0.885, *T*_max_ = 0.908
36292 measured reflections
7115 independent reflections
4248 reflections with *I* > 2σ(*I*)
*R*_int_ = 0.058

#### Refinement

*R*[*F* ^2^ > 2σ(*F* ^2^)] = 0.038
*wR*(*F* ^2^) = 0.093
*S* = 1.00
7115 reflections
424 parameters
H-atom parameters constrained
Δρ_max_ = 0.25 e Å^−3^
Δρ_min_ = −0.26 e Å^−3^

### 

Data collection: *SMART* (Bruker, 1997 ▶); cell refinement: *SAINT* (Bruker, 1997 ▶); data reduction: *SAINT*; program(s) used to solve structure: *SHELXS97* (Sheldrick, 2008 ▶); program(s) used to refine structure: *SHELXL97* (Sheldrick, 2008 ▶); molecular graphics: *SHELXTL* (Sheldrick, 2008 ▶); software used to prepare material for publication: *publCIF* (Westrip, 2008 ▶).

## Supplementary Material

Crystal structure: contains datablocks I, global. DOI: 10.1107/S1600536808023489/is2292sup1.cif

Structure factors: contains datablocks I. DOI: 10.1107/S1600536808023489/is2292Isup2.hkl

Additional supplementary materials:  crystallographic information; 3D view; checkCIF report

## Figures and Tables

**Fig. 1.**
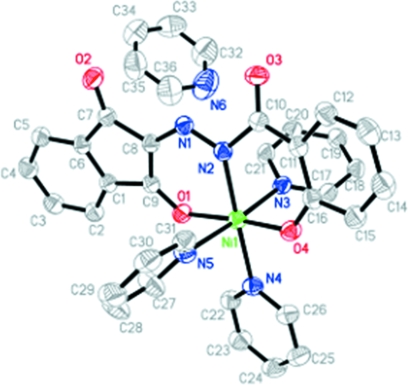
The molecular structure of the title compound, showing the atom-labelling scheme. Displacement ellipsoids are drawn at the 30% probability level. H atoms have been omitted for clarity.

**Fig. 2.**
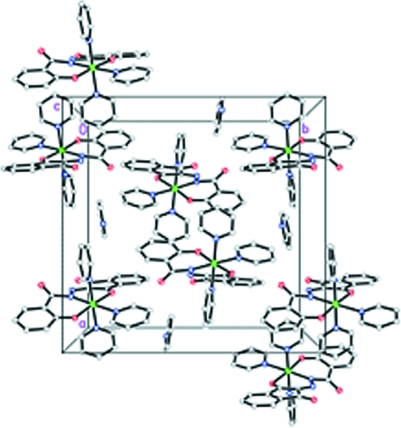
A partial packing diagram of the title compound.

## supplementary crystallographic information

### Comment

As part of an ongoing study on the structural characterization of Schiff-base compounds (Qiu, Fang *et al.*, 2006; Qiu, Luo *et al.*, 2006), the crystal structure of the title compound is reported here. In the molecule (Fig. 1), the Ni^II^ ion is six-coordinated by two oxygen atoms and one nitrogen atom from the schiff base ligand and three nitrogen atoms from three pyridine rings. One pyridine solvent molecule is not involved either in coordination to the Ni^II^ center or in classic hydrogen bonding to the compound. The Ni–O (phenolate) bond (1.975 Å) is the significantly shorter than other Ni–O (carbonyl) (2.084 Å) and Ni–N bonds (mean 2.120 Å), which suggests that the Ni–O (phenolate) bond is stronger than other bonds. From the crystal structure, the schiff base ligand and pyridine rings wrap around the Ni^II^ centre, forming an octahedral coordination (Qiu *et al.*, 2007). A portion of the crystal packing of the compound is illustrated in Fig. 2.

### Experimental

To a cold solution of 2-hydroxybenzhydrazide (3.04 g, 20 mmol) in absolute ethyl alcohol (25 ml) was added dropwise a solution of triketohydrindene hydrate (3.2 g, 20 mmol) in absolute ethyl alcohol (25 ml). Stirring was continued at room temperature for 10 min, then refluxing at 351 K for 2 h. After filtering, the filtrate was the schiff base (H_2_L) as yellow solid. To a solution of ligand (0.47 g, 1.6 mmol) in ethyl acetate (15 ml) was added slowly a solution of Ni(*ac*)_2_.2H_2_O (0.34 g, 1.6 mmol) in ethyl acetate (10 ml). The mixture was stirred for 2 h until a brown precipitate appeared. The precipitate was collected and washed three times with ethyl acetate. Further drying in vacuum afforded a brown powder. Brown single crystals of NiL.4(C_5_H_5_N) were grown from methanol and pyridine mixed solution (2:1 *v*/*v*) with slow evaporation at room temperature.

### Refinement

All H were placed in geometrically idealized positions (C—H = 0.93 Å) and were treated as riding atoms, with *U*_iso_(H) = 1.2*U*_eq_(C).

### Crystal data

**Table:**

[Ni(C_16_H_8_N_2_O_4_)(C_5_H_5_N)_3_]·C_5_H_5_N	*F*_000_ = 2768
*M**_r_* = 667.35	*D*_x_ = 1.358 Mg m^−^^3^
Orthorhombic, *P**b**c**a*	Mo *K*α radiation λ = 0.71073 Å
Hall symbol: -P 2ac 2ab	Cell parameters from 4952 reflections
*a* = 17.1945 (13) Å	θ = 2.2–21.5º
*b* = 17.6887 (13) Å	µ = 0.64 mm^−^^1^
*c* = 21.4633 (16) Å	*T* = 273 (2) K
*V* = 6528.0 (8) Å^3^	Block, brown
*Z* = 8	0.19 × 0.18 × 0.15 mm

### Data collection

**Table:**

Bruker SMART 1000 CCD area-detector diffractometer	7115 independent reflections
Radiation source: fine-focus sealed tube	4248 reflections with *I* > 2σ(*I*)
Monochromator: graphite	*R*_int_ = 0.058
*T* = 273(2) K	θ_max_ = 27.0º
φ and ω scans	θ_min_ = 1.9º
Absorption correction: multi-scan(*SADABS*; Sheldrick, 1996)	*h* = −20→21
*T*_min_ = 0.885, *T*_max_ = 0.908	*k* = −18→22
36292 measured reflections	*l* = −24→27

### Refinement

**Table:**

Refinement on *F*^2^	Secondary atom site location: difference Fourier map
Least-squares matrix: full	Hydrogen site location: inferred from neighbouring sites
*R*[*F*^2^ > 2σ(*F*^2^)] = 0.038	H-atom parameters constrained
*wR*(*F*^2^) = 0.093	*w* = 1/[σ^2^(*F*_o_^2^) + (0.031*P*)^2^ + 1.8991*P*] where *P* = (*F*_o_^2^ + 2*F*_c_^2^)/3
*S* = 1.00	(Δ/σ)_max_ = 0.001
7115 reflections	Δρ_max_ = 0.25 e Å^−^^3^
424 parameters	Δρ_min_ = −0.26 e Å^−^^3^
Primary atom site location: structure-invariant direct methods	Extinction correction: none

### Special details

**Table:**

Geometry. All e.s.d.'s (except the e.s.d. in the dihedral angle between two l.s. planes) are estimated using the full covariance matrix. The cell e.s.d.'s are taken into account individually in the estimation of e.s.d.'s in distances, angles and torsion angles; correlations between e.s.d.'s in cell parameters are only used when they are defined by crystal symmetry. An approximate (isotropic) treatment of cell e.s.d.'s is used for estimating e.s.d.'s involving l.s. planes.
Refinement. Refinement of *F*^2^ against ALL reflections. The weighted *R*-factor *wR* and goodness of fit *S* are based on *F*^2^, conventional *R*-factors *R* are based on *F*, with *F* set to zero for negative *F*^2^. The threshold expression of *F*^2^ > σ(*F*^2^) is used only for calculating *R*-factors(gt) *etc*. and is not relevant to the choice of reflections for refinement. *R*-factors based on *F*^2^ are statistically about twice as large as those based on *F*, and *R*- factors based on ALL data will be even larger.

### Fractional atomic coordinates and isotropic or equivalent isotropic displacement parameters (Å^2^)

**Table:**

	*x*	*y*	*z*	*U*_iso_*/*U*_eq_	
Ni1	0.836338 (16)	0.083641 (16)	0.657583 (13)	0.04341 (10)	
C1	0.76080 (12)	0.18336 (13)	0.48329 (11)	0.0453 (6)	
C2	0.75643 (14)	0.26298 (14)	0.47940 (12)	0.0565 (7)	
H2	0.7638	0.2930	0.5145	0.068*	
C3	0.74079 (15)	0.29530 (16)	0.42153 (13)	0.0643 (7)	
H3	0.7383	0.3476	0.4174	0.077*	
C4	0.72894 (15)	0.24950 (17)	0.37029 (13)	0.0668 (8)	
H4	0.7185	0.2719	0.3320	0.080*	
C5	0.73212 (14)	0.16902 (15)	0.37408 (12)	0.0595 (7)	
H5	0.7235	0.1393	0.3390	0.071*	
C6	0.74836 (13)	0.13645 (14)	0.43112 (11)	0.0463 (6)	
C7	0.75640 (13)	0.05326 (14)	0.44978 (11)	0.0476 (6)	
C8	0.77700 (13)	0.05377 (13)	0.51617 (10)	0.0431 (5)	
C9	0.77869 (12)	0.13320 (13)	0.53733 (11)	0.0433 (6)	
C10	0.80145 (16)	−0.08939 (15)	0.63042 (12)	0.0568 (7)	
C11	0.84936 (14)	−0.11063 (14)	0.68389 (11)	0.0489 (6)	
C12	0.85576 (15)	−0.19104 (15)	0.69505 (13)	0.0615 (7)	
H12	0.8306	−0.2240	0.6680	0.074*	
C13	0.89690 (16)	−0.22044 (16)	0.74336 (14)	0.0693 (8)	
H13	0.9009	−0.2724	0.7493	0.083*	
C14	0.93282 (16)	−0.16944 (17)	0.78369 (14)	0.0692 (8)	
H14	0.9608	−0.1880	0.8175	0.083*	
C15	0.92794 (14)	−0.09100 (15)	0.77480 (12)	0.0590 (7)	
H15	0.9527	−0.0593	0.8031	0.071*	
C16	0.88692 (13)	−0.05805 (14)	0.72444 (11)	0.0470 (6)	
C17	0.72290 (15)	0.05699 (15)	0.76300 (11)	0.0568 (7)	
H17	0.7682	0.0546	0.7866	0.068*	
C18	0.65464 (16)	0.04440 (16)	0.79189 (12)	0.0644 (7)	
H18	0.6528	0.0336	0.8343	0.077*	
C19	0.58917 (15)	0.04784 (15)	0.75774 (12)	0.0616 (7)	
H19	0.5409	0.0396	0.7761	0.074*	
C20	0.59477 (14)	0.06369 (16)	0.69538 (13)	0.0630 (7)	
H20	0.5502	0.0660	0.6709	0.076*	
C21	0.66456 (14)	0.07584 (15)	0.67011 (11)	0.0579 (7)	
H21	0.6675	0.0870	0.6278	0.069*	
C22	0.84087 (14)	0.25500 (15)	0.69829 (12)	0.0577 (7)	
H22	0.8132	0.2617	0.6615	0.069*	
C23	0.85674 (17)	0.31949 (16)	0.73437 (14)	0.0702 (8)	
H23	0.8403	0.3672	0.7217	0.084*	
C24	0.89680 (17)	0.31003 (18)	0.78832 (15)	0.0756 (9)	
H24	0.9084	0.3511	0.8137	0.091*	
C25	0.92010 (16)	0.23715 (18)	0.80478 (14)	0.0720 (8)	
H25	0.9478	0.2295	0.8414	0.086*	
C26	0.90217 (15)	0.17552 (16)	0.76671 (12)	0.0619 (7)	
H26	0.9180	0.1275	0.7789	0.074*	
C27	0.96595 (17)	0.15987 (18)	0.58237 (15)	0.0824 (10)	
H27	0.9369	0.2034	0.5893	0.099*	
C28	1.0323 (2)	0.1649 (2)	0.54845 (18)	0.1044 (12)	
H28	1.0491	0.2109	0.5324	0.125*	
C29	1.07387 (18)	0.0991 (2)	0.53864 (16)	0.0895 (11)	
H29	1.1197	0.1008	0.5157	0.107*	
C30	1.04850 (16)	0.0314 (2)	0.56227 (13)	0.0723 (9)	
H30	1.0762	−0.0130	0.5551	0.087*	
C31	0.98246 (14)	0.03074 (16)	0.59608 (11)	0.0572 (7)	
H31	0.9652	−0.0148	0.6128	0.069*	
C32	0.4254 (3)	0.1039 (2)	0.5441 (2)	0.1206 (14)	
H32	0.3875	0.0997	0.5749	0.145*	
C33	0.4032 (2)	0.0948 (2)	0.4840 (2)	0.1056 (12)	
H33	0.3512	0.0867	0.4739	0.127*	
C34	0.4572 (3)	0.0977 (2)	0.43937 (19)	0.1094 (13)	
H34	0.4443	0.0909	0.3977	0.131*	
C35	0.5290 (3)	0.1106 (2)	0.4563 (2)	0.1157 (14)	
H35	0.5682	0.1125	0.4265	0.139*	
C36	0.5462 (2)	0.1208 (3)	0.5161 (2)	0.1186 (14)	
H36	0.5977	0.1308	0.5263	0.142*	
N1	0.78470 (10)	−0.01309 (11)	0.54711 (9)	0.0448 (5)	
N2	0.80692 (10)	−0.01229 (10)	0.60518 (9)	0.0441 (5)	
N3	0.72906 (11)	0.07276 (11)	0.70230 (9)	0.0464 (5)	
N4	0.86311 (11)	0.18326 (11)	0.71342 (9)	0.0496 (5)	
N5	0.94071 (11)	0.09374 (12)	0.60636 (9)	0.0538 (5)	
N6	0.4958 (2)	0.1180 (2)	0.56149 (16)	0.1264 (12)	
O1	0.79103 (9)	0.15745 (8)	0.59117 (7)	0.0493 (4)	
O2	0.74742 (11)	−0.00276 (10)	0.41554 (8)	0.0645 (5)	
O3	0.75743 (14)	−0.13547 (12)	0.60754 (9)	0.0958 (8)	
O4	0.88726 (9)	0.01748 (9)	0.71947 (7)	0.0523 (4)	

### Atomic displacement parameters (Å^2^)

**Table:**

	*U*^11^	*U*^22^	*U*^33^	*U*^12^	*U*^13^	*U*^23^
Ni1	0.03906 (16)	0.04585 (18)	0.04532 (18)	−0.00026 (14)	0.00063 (14)	−0.00265 (15)
C1	0.0367 (13)	0.0499 (16)	0.0493 (14)	0.0019 (11)	0.0044 (10)	0.0021 (12)
C2	0.0575 (16)	0.0531 (17)	0.0589 (17)	0.0006 (13)	0.0035 (13)	−0.0007 (14)
C3	0.0672 (19)	0.0513 (17)	0.074 (2)	0.0031 (14)	−0.0003 (15)	0.0094 (16)
C4	0.0692 (18)	0.066 (2)	0.0651 (18)	0.0006 (15)	−0.0071 (14)	0.0201 (16)
C5	0.0590 (17)	0.069 (2)	0.0506 (16)	−0.0045 (14)	−0.0020 (12)	0.0041 (14)
C6	0.0387 (13)	0.0524 (15)	0.0479 (15)	0.0011 (11)	0.0044 (11)	0.0037 (12)
C7	0.0434 (14)	0.0521 (15)	0.0472 (14)	0.0000 (12)	0.0034 (11)	−0.0017 (13)
C8	0.0399 (13)	0.0467 (14)	0.0425 (13)	0.0003 (11)	0.0029 (10)	0.0018 (12)
C9	0.0339 (12)	0.0486 (15)	0.0473 (14)	0.0019 (10)	0.0059 (10)	0.0001 (12)
C10	0.0708 (17)	0.0490 (16)	0.0506 (15)	−0.0109 (14)	−0.0028 (13)	0.0008 (13)
C11	0.0497 (15)	0.0473 (15)	0.0496 (14)	0.0008 (12)	0.0025 (12)	0.0044 (12)
C12	0.0693 (19)	0.0509 (17)	0.0644 (18)	−0.0017 (14)	0.0060 (14)	0.0030 (14)
C13	0.071 (2)	0.0537 (18)	0.084 (2)	0.0101 (15)	0.0059 (17)	0.0166 (16)
C14	0.0566 (18)	0.075 (2)	0.076 (2)	0.0088 (15)	−0.0065 (15)	0.0199 (17)
C15	0.0512 (16)	0.0662 (19)	0.0596 (16)	0.0008 (14)	−0.0077 (12)	0.0094 (14)
C16	0.0384 (13)	0.0521 (17)	0.0506 (15)	0.0022 (11)	0.0051 (11)	0.0052 (12)
C17	0.0488 (15)	0.0742 (18)	0.0472 (15)	−0.0015 (13)	−0.0017 (12)	−0.0037 (14)
C18	0.0584 (18)	0.090 (2)	0.0445 (15)	−0.0034 (15)	0.0081 (13)	0.0028 (15)
C19	0.0471 (16)	0.0785 (19)	0.0590 (18)	−0.0057 (14)	0.0125 (13)	0.0006 (15)
C20	0.0393 (15)	0.090 (2)	0.0598 (18)	−0.0058 (14)	−0.0023 (13)	−0.0021 (15)
C21	0.0449 (14)	0.084 (2)	0.0444 (14)	−0.0048 (14)	0.0024 (12)	0.0023 (13)
C22	0.0574 (16)	0.0549 (17)	0.0607 (16)	0.0017 (14)	0.0018 (13)	−0.0073 (14)
C23	0.079 (2)	0.0539 (18)	0.077 (2)	0.0009 (15)	0.0049 (17)	−0.0124 (16)
C24	0.077 (2)	0.071 (2)	0.078 (2)	−0.0140 (17)	0.0031 (17)	−0.0286 (18)
C25	0.0667 (19)	0.082 (2)	0.0669 (19)	−0.0056 (17)	−0.0090 (15)	−0.0162 (17)
C26	0.0552 (17)	0.0651 (19)	0.0653 (18)	−0.0018 (13)	−0.0048 (14)	−0.0078 (15)
C27	0.068 (2)	0.067 (2)	0.111 (3)	−0.0087 (16)	0.0337 (19)	−0.0041 (18)
C28	0.088 (3)	0.089 (3)	0.135 (3)	−0.029 (2)	0.051 (2)	−0.010 (2)
C29	0.0535 (19)	0.123 (3)	0.092 (2)	−0.020 (2)	0.0220 (17)	−0.035 (2)
C30	0.0428 (16)	0.103 (3)	0.071 (2)	0.0046 (16)	−0.0009 (14)	−0.0283 (18)
C31	0.0444 (15)	0.0712 (19)	0.0559 (16)	0.0044 (13)	−0.0039 (12)	−0.0083 (14)
C32	0.090 (3)	0.160 (4)	0.112 (4)	0.017 (3)	0.015 (3)	−0.011 (3)
C33	0.074 (3)	0.103 (3)	0.140 (4)	0.010 (2)	−0.021 (3)	−0.026 (3)
C34	0.114 (4)	0.126 (3)	0.088 (3)	0.008 (3)	−0.011 (3)	−0.011 (2)
C35	0.096 (3)	0.156 (4)	0.095 (3)	0.007 (3)	0.012 (2)	0.022 (3)
C36	0.071 (3)	0.166 (4)	0.118 (4)	0.012 (3)	−0.012 (3)	0.030 (3)
N1	0.0429 (11)	0.0495 (13)	0.0420 (12)	−0.0005 (9)	0.0027 (9)	−0.0022 (10)
N2	0.0408 (11)	0.0471 (12)	0.0444 (12)	−0.0013 (9)	0.0017 (9)	0.0015 (9)
N3	0.0412 (11)	0.0555 (13)	0.0424 (11)	−0.0022 (9)	0.0005 (9)	−0.0039 (10)
N4	0.0406 (11)	0.0523 (14)	0.0559 (13)	−0.0005 (10)	0.0010 (10)	−0.0066 (10)
N5	0.0424 (12)	0.0587 (14)	0.0601 (13)	−0.0036 (11)	0.0046 (10)	−0.0075 (11)
N6	0.094 (3)	0.190 (4)	0.096 (2)	0.019 (3)	−0.019 (2)	0.000 (2)
O1	0.0529 (10)	0.0490 (10)	0.0462 (10)	0.0026 (8)	0.0000 (8)	−0.0047 (8)
O2	0.0869 (13)	0.0572 (11)	0.0494 (10)	0.0001 (10)	−0.0032 (9)	−0.0066 (9)
O3	0.145 (2)	0.0689 (13)	0.0739 (14)	−0.0529 (14)	−0.0469 (13)	0.0201 (11)
O4	0.0517 (10)	0.0515 (11)	0.0537 (10)	0.0002 (8)	−0.0100 (8)	0.0003 (8)

### Geometric parameters (Å, °)

**Table:**

Ni1—O1	2.0840 (16)	C18—H18	0.9300
Ni1—O4	1.9750 (16)	C19—C20	1.371 (3)
Ni1—N2	2.0977 (19)	C19—H19	0.9300
Ni1—N3	2.0882 (18)	C20—C21	1.334 (3)
Ni1—N4	2.180 (2)	C20—H20	0.9300
Ni1—N5	2.1122 (19)	C21—N3	1.308 (3)
C1—C2	1.413 (3)	C21—H21	0.9300
C1—C6	1.410 (3)	C22—N4	1.365 (3)
C1—C9	1.492 (3)	C22—C23	1.405 (3)
C2—C3	1.394 (3)	C22—H22	0.9300
C2—H2	0.9300	C23—C24	1.358 (4)
C3—C4	1.381 (4)	C23—H23	0.9300
C3—H3	0.9300	C24—C25	1.395 (4)
C4—C5	1.427 (4)	C24—H24	0.9300
C4—H4	0.9300	C25—C26	1.397 (4)
C5—C6	1.382 (3)	C25—H25	0.9300
C5—H5	0.9300	C26—N4	1.333 (3)
C6—C7	1.531 (3)	C26—H26	0.9300
C7—O2	1.243 (3)	C27—N5	1.350 (3)
C7—C8	1.468 (3)	C27—C28	1.356 (4)
C8—N1	1.363 (3)	C27—H27	0.9300
C8—C9	1.477 (3)	C28—C29	1.382 (4)
C9—O1	1.251 (3)	C28—H28	0.9300
C10—O3	1.216 (3)	C29—C30	1.373 (4)
C10—C11	1.462 (3)	C29—H29	0.9300
C10—N2	1.471 (3)	C30—C31	1.348 (3)
C11—C16	1.428 (3)	C30—H30	0.9300
C11—C12	1.447 (3)	C31—N5	1.344 (3)
C12—C13	1.359 (4)	C31—H31	0.9300
C12—H12	0.9300	C32—N6	1.292 (5)
C13—C14	1.394 (4)	C32—C33	1.357 (5)
C13—H13	0.9300	C32—H32	0.9300
C14—C15	1.403 (4)	C33—C34	1.335 (5)
C14—H14	0.9300	C33—H33	0.9300
C15—C16	1.416 (3)	C34—C35	1.307 (5)
C15—H15	0.9300	C34—H34	0.9300
C16—O4	1.340 (3)	C35—C36	1.329 (5)
C17—N3	1.337 (3)	C35—H35	0.9300
C17—C18	1.346 (3)	C36—N6	1.303 (5)
C17—H17	0.9300	C36—H36	0.9300
C18—C19	1.345 (3)	N1—N2	1.304 (2)
			
O4—Ni1—O1	175.52 (7)	C18—C19—H19	120.6
O4—Ni1—N3	91.60 (7)	C20—C19—H19	120.6
O1—Ni1—N3	92.40 (7)	C21—C20—C19	119.5 (2)
O4—Ni1—N2	89.32 (7)	C21—C20—H20	120.2
O1—Ni1—N2	92.86 (7)	C19—C20—H20	120.2
N3—Ni1—N2	87.64 (7)	N3—C21—C20	122.8 (2)
O4—Ni1—N5	91.34 (7)	N3—C21—H21	118.6
O1—Ni1—N5	84.76 (7)	C20—C21—H21	118.6
N3—Ni1—N5	175.99 (8)	N4—C22—C23	124.7 (3)
N2—Ni1—N5	89.67 (7)	N4—C22—H22	117.7
O4—Ni1—N4	90.90 (7)	C23—C22—H22	117.7
O1—Ni1—N4	87.05 (7)	C24—C23—C22	117.9 (3)
N3—Ni1—N4	90.48 (7)	C24—C23—H23	121.0
N2—Ni1—N4	178.12 (7)	C22—C23—H23	121.0
N5—Ni1—N4	92.20 (7)	C23—C24—C25	118.4 (3)
C6—C1—C2	122.1 (2)	C23—C24—H24	120.8
C6—C1—C9	107.4 (2)	C25—C24—H24	120.8
C2—C1—C9	130.5 (2)	C24—C25—C26	120.6 (3)
C3—C2—C1	118.2 (3)	C24—C25—H25	119.7
C3—C2—H2	120.9	C26—C25—H25	119.7
C1—C2—H2	120.9	N4—C26—C25	122.2 (3)
C4—C3—C2	119.8 (3)	N4—C26—H26	118.9
C4—C3—H3	120.1	C25—C26—H26	118.9
C2—C3—H3	120.1	N5—C27—C28	122.1 (3)
C3—C4—C5	122.3 (3)	N5—C27—H27	118.9
C3—C4—H4	118.9	C28—C27—H27	118.9
C5—C4—H4	118.9	C27—C28—C29	117.5 (3)
C6—C5—C4	118.3 (3)	C27—C28—H28	121.2
C6—C5—H5	120.8	C29—C28—H28	121.2
C4—C5—H5	120.8	C30—C29—C28	120.9 (3)
C5—C6—C1	119.3 (2)	C30—C29—H29	119.5
C5—C6—C7	130.6 (2)	C28—C29—H29	119.5
C1—C6—C7	110.1 (2)	C31—C30—C29	118.3 (3)
O2—C7—C8	127.5 (2)	C31—C30—H30	120.8
O2—C7—C6	126.9 (2)	C29—C30—H30	120.8
C8—C7—C6	105.6 (2)	N5—C31—C30	122.1 (3)
N1—C8—C7	119.4 (2)	N5—C31—H31	118.9
N1—C8—C9	132.4 (2)	C30—C31—H31	118.9
C7—C8—C9	108.0 (2)	N6—C32—C33	124.1 (4)
O1—C9—C8	127.9 (2)	N6—C32—H32	117.9
O1—C9—C1	123.3 (2)	C33—C32—H32	117.9
C8—C9—C1	108.82 (19)	C34—C33—C32	118.8 (4)
O3—C10—C11	119.7 (2)	C34—C33—H33	120.6
O3—C10—N2	120.8 (2)	C32—C33—H33	120.6
C11—C10—N2	119.4 (2)	C35—C34—C33	117.7 (4)
C16—C11—C12	120.3 (2)	C35—C34—H34	121.2
C16—C11—C10	124.5 (2)	C33—C34—H34	121.2
C12—C11—C10	115.2 (2)	C34—C35—C36	120.1 (4)
C13—C12—C11	122.8 (3)	C34—C35—H35	119.9
C13—C12—H12	118.6	C36—C35—H35	119.9
C11—C12—H12	118.6	N6—C36—C35	124.7 (4)
C12—C13—C14	117.2 (3)	N6—C36—H36	117.7
C12—C13—H13	121.4	C35—C36—H36	117.7
C14—C13—H13	121.4	N2—N1—C8	119.01 (19)
C13—C14—C15	121.9 (3)	N1—N2—C10	108.86 (18)
C13—C14—H14	119.0	N1—N2—Ni1	126.31 (15)
C15—C14—H14	119.0	C10—N2—Ni1	124.62 (15)
C14—C15—C16	122.7 (3)	C21—N3—C17	117.2 (2)
C14—C15—H15	118.6	C21—N3—Ni1	120.16 (16)
C16—C15—H15	118.6	C17—N3—Ni1	122.50 (16)
O4—C16—C15	118.0 (2)	C26—N4—C22	116.2 (2)
O4—C16—C11	127.1 (2)	C26—N4—Ni1	119.66 (18)
C15—C16—C11	115.0 (2)	C22—N4—Ni1	124.17 (17)
N3—C17—C18	123.6 (2)	C31—N5—C27	119.0 (2)
N3—C17—H17	118.2	C31—N5—Ni1	117.98 (17)
C18—C17—H17	118.2	C27—N5—Ni1	123.02 (18)
C19—C18—C17	118.1 (2)	C32—N6—C36	114.5 (4)
C19—C18—H18	120.9	C9—O1—Ni1	118.72 (15)
C17—C18—H18	120.9	C16—O4—Ni1	129.97 (15)
C18—C19—C20	118.9 (2)		
